# Spectrophotometric Estimation of Ropinirole Hydrochloride in Tablets

**DOI:** 10.4103/0250-474X.51962

**Published:** 2009

**Authors:** Yogita Shete, Nayana Pimpodkar, R. S. Nalawade, Y. V. Pore, B. S. Kuchekar

**Affiliations:** Government College of Pharmacy, Vidyanagar, Karad-415 214, India

**Keywords:** Ropinirole hydrochloride, spectrophotometric estimation, tablets

## Abstract

A simple, sensitive, rapid, accurate and precise spectrophotometric method has been developed for estimation of ropinirole hydrochloride in bulk and tablet dosage forms. Ropinirole hydrochloride shows maximum absorbance at 250 nm with molar absorptivity of 8.703×10^3^ l/mol.cm. Beer's law was obeyed in the concentration range of 5-35 μg/ml. Results of analysis were validated statistically and by recovery studies.

Ropinirole hydrochloride, chemically known as 4[2-(dipropylamine ethyl)]-1,3-dihydro-2H indole-2-one monohydrochloride, is a non-ergot dopamine D2-antagonist with similar actions to those of bromocriptine[1,2]. It is used as antiparkinson agent[3,4]. Only HPLC and thermospray liquid chromatography are reported for estimation ropinirole hydrochloride in formulation[3,4].

The objective of the study was to develop a simple, rapid, accurate and specific spectrophotometric method for the estimation of ropinirole hydrochloride using UV spectrophotometry. The simple method was developed using distilled water as a solvent with minimum processing steps. The λ_max_ of ropinirole in distilled water was found to be 250 nm and Beer's law was obeyed in the range of 5-35 μg/ml. The result of analysis was validated statistically using recovery studies. Thus this method of estimation of ropinirole was found to be simple, precise and accurate.

A Shimadzu 1700 UV spectrophotometer with 1 cm matched cuvettes were used for estimation. Standard solution of drug (100 μg/ml) was prepared in distilled water. Twenty tablets of ropinirole hydrochloride were weighed and powdered in glass mortar. Powder equivalent to 10 mg of the drug was transferred to 100 ml volumetric flask, dissolved in about 50ml distilled water and made up the volume to the mark with distilled water to obtain the concentration of 100 μg/ml.

Aliquots of 0.5 to 3.5 ml portions of the standard solution were transferred to a series of calibrated 10 ml corning test tubes and the volume in each test tube was adjusted to 10 ml with distilled water. The absorbance of solutions was measured at 250 nm against reagent blank and calibration curve was constructed. Similarly absorbance of sample solution was measured and amount of ropinirole hydrochloride was determined by referring to the calibration curve. Recovery studies were carried out by adding a known quantity of the pure drug to the preanalyzed formulation and the proposed method was followed. From the amount of drug found, percentage recovery was calculated.

The proposed method of determination of ropinirole hydrochloride showed molar absorptivity of 8.703×10^3^ l/mol.cm and Sandell's sensitivity 0.0341 μg/cm^2^/0.001-absorbance units. Linear regression of absorbance Vs concentration yielded equation y=0.0293x+0.00357 with a correlation coefficient of 0.9998. Relative standard deviation of 0.00608 was observed for analysis of three replicate samples, indicating precision and reproducibility.

Ropinirole hydrochloride exhibits its maximum absorption at 250 nm and obeyed Beer's law in the range of 5-35 μg/ml. The results of analysis and recovery studies are presented in the Table 1. The percentage recovery value 99%-101% indicates that there is no interference from the excipients present in the formulation. The developed method was found to be sensitive, accurate, precise and reproducible and can be used for the routine quality control analysis of ropinirole hydrochloride in bulk drugs and formulations.

**TABLE 1:** RESULTS OF ANALYSIS AND RECOVERY STUDIES

Formulations	Label Claim	% Estimated	SD	COV (%)	SE	% Recovery
Lab mixture	5	99.39	0.60	0.61	0.35	100.61

SD is standard deviation, SE is standard error and COV is coefficient of variation

## References

[1] Budavari S (1996). The Merck Index.

[2] Reynolds JEF (1993). Martindale; the Extra Pharmacopoeia.

[3] Coufal P, Stulik K, Claessens HA, Harday MJ, Webb M (1999). Separation and quantification of ropinirole and some impurities using capillary liquid chromatography. J Chromatogr Biomed Sci Appl.

[4] Beattie IG, Blake TJ (1989). Application of thermospray liquid chromatography-mass spectrometry and liquid chromatography-tandem mass spectrometry for the identification of cynomolgus monkey and human metabolities of SK&F 101468, a dopamine D_2_ receptor agonist. J Chromatogr.

